# Endometrial cells with high ALDH activity contribute to uterine development and regeneration

**DOI:** 10.21203/rs.3.rs-6831916/v3

**Published:** 2026-05-26

**Authors:** Suni Tang, Anna Catherine Unser, Peixin Jiang, Sydney E. Parks, Genesis J. Herrera, Ting Geng, Linda Alpuing Radilla, Brooke A. Thigpen, Xiaoming Guan, Diana Monsivais

**Affiliations:** 1 Department of Pathology & Immunology, Baylor College of Medicine, Houston, Texas, USA; 2 Center for Drug Discovery, Baylor College of Medicine, Houston, Texas, USA; 3 Department of Obstetrics and Gynecology, Baylor College of Medicine, Houston, Texas, USA

**Keywords:** Lineage tracing, organoid, endometrium, stem cells, endometriosis, inflammation, progenitors

## Abstract

Adult stem cells are thought to drive the regenerative potential of the endometrium and contribute to the pathogenesis of endometriosis, however, their identity and defining features remain to be characterized. Here, we used *in vivo* and *in vitro* approaches to demonstrate that cells with high aldehyde dehydrogenase 1 activity (ALDH^HI^ cells) were long lived progenitors in the endometrium with a higher organoid formation capacity, long-term passaging potential, and stemness gene signatures. Using lineage tracing with an *Aldh1a1*^*cre/ERT2*^; *ROSA26*^*tdTomato*^ reporter mouse, *Aldh1a1*^+^ epithelial cells expanded during postnatal development, *Aldh1a1*^+^ stromal cells expanded during estrous cycling, and both populations of *Aldh1a1*^+^ cells were present during postpartum repair. In response to ovariectomy or exogenous estradiol, we found that ALDH1A1^+^ cells localized to glandular crypts of the endometrium or throughout the luminal epithelium, respectively, indicating that their spatial localization is hormone sensitive. Functionally, we found that selective ablation of ALDH1A1^+^ cells in *Aldh1a1*^*cre/ERT2*^; *ROSA26*-DTR^flox/flox^ mice decreased endometrial gland number and FOXA2 expression. These findings were recapitulated in the human endometrium, where endometrial epithelial organoids with high ALDH activity (ALDH^HI^ cells) showed a higher organoid formation capacity than ALDH^LO^ cells and displayed unique transcriptomes with fewer luminal-like ciliated cells. Overall, our studies indicate that ALDH1A1^+^ cells are hormone-sensitive adult stem cells in the endometrium with regenerative potential that are critical for endometrial development and function.

## Brief Summary

We uncover ALDH1A1^+^ cells as a group of hormone sensitive stem cells contributing to endometrial development and regeneration.

## Introduction

The endometrium is the inner lining of the uterine cavity whose unique regenerative potential is fueled by stem cells (Cousins et al., 2021). The location and identity of these adult stem cells is an active area of investigation due to the high prevalence of endometriosis, adenomyosis, and other menstrual-related pathologies that arise from endometrial tissue. Recent studies have used classical stem cell assays to identify *CDH2*^+^, *SSEA1*^+^, and *SUSD2*^+^ progenitors that are enriched in the basalis endometrium and localize to the perivascular regions of the endometrium (Masuda et al., 2012; Nguyen et al., 2017; Valentijn et al., 2013). Using these markers, several groups have detected *CDH2*^+^, *SSEA1*^+^, SOX9^+^, and *SUSD2*^+^ stem cells in the menstrual effluent and peritoneal fluid of women with endometriosis (Hapangama et al., 2019; Masuda et al., 2021). These markers identify specific cell types in the endometrium, with *CDH2*^+^, *SSEA1*^+^, and *SOX9*^+^ cells corresponding to endometrial epithelial cells, while *SUSD2*^+^ cells corresponding to mesenchymal endometrial cells enriched in the perivascular regions (Cousins et al., 2021). These studies support the hypothesis that stem cells present in menstrual effluent enter the peritoneal cavity during retrograde menstruation and are a key factor in the establishment of endometriosis (Bulun, 2022; Sampson, 1927). Therefore, defining stem cell identity and signaling networks within the eutopic endometrium is key to developing novel targeted approaches for the treatment of ectopic endometriotic lesions and their associated pain, inflammation, and infertility.

The mouse has been used to identify stem cell markers driving the regenerative potential of the endometrium. Specifically, lineage tracing studies identified a putative stem cell niche that is enriched at the junctional zone between the luminal and glandular epithelium in the endometrium (Jin, 2019). Studies tracing the fate, ablation, and proliferative capacity of *Lgr5*^+^ cells in the uterus identified an *Lgr5*^+^ niche that marked stem/progenitor cells in the developing uterus (Seishima et al., 2019). Similarly, using lineage tracing, ablation, and organoid models, *Axin2*^+^ cells were shown to be localized within the glandular crypts of the mouse endometrium, driving normal homeostasis and oncogenic transformation in the endometrium (Syed et al., 2020).

Maintenance of endometrial architecture during the estrous cycle and the structural remodeling of the postpartum period involves the coordinated expansion and differentiation of stem cells. We identified that conditional inactivation of the TGFβ receptor, ALK5, impairs postpartum endometrial remodeling, leading to structural defects, tumors, and lung metastases (Monsivais et al., 2019). We also showed that conditional inactivation of the downstream effectors of TGFβ, the SMAD2 and SMAD3 transcription factors, resulted in aggressive and metastatic endometrial tumors (Kriseman et al., 2019; Kriseman et al., 2023). When cultured *in vitro,* organoids derived from mice with conditional SMAD2/3 inactivation, as well as control organoids treated with A83–01 (an ALK4/5/7 inhibitor), developed an altered morphology and elevated expression of retinoic acid signaling molecules, including elevated expression of aldehyde dehydrogenase 1a1 (*Aldh1a1*) (Kriseman et al., 2023) (Tojo et al., 2005). Furthermore, endometrial epithelial organoids from mice lacking TGFBR2 become stratified and abnormally express keratin 5 and p63, highlighting its critical role in epithelial cell type specification (Parks et al., 2025). Thus, disruption of TGFβ signaling impaired organoid and endometrial homeostasis through alterations in the retinoic acid pathway.

One important enzyme in retinoic acid metabolism is aldehyde dehydrogenase 1a1 (ALDH1A1), which catalyzes the conversion of retinaldehyde into retinoic acid (Haselbeck et al., 1999). Previous studies suggested that high expression and activity of ALDH1A1 is a putative stemness marker in the endometrium of women and mice (Wu et al., 2017). In the postnatal mouse endometrium, ALDH1A1 is abundantly expressed throughout the epithelium suggesting a role in developmental uterine maturation (Spencer et al., 2023). In humans, ALDH1A1 is enriched in the basalis endometrial epithelium, where it colocalizes with endometrial stem cell marker CDH2 and is expressed in ectopic endometriotic lesions on the ovary (Ma et al., 2020). Here, we used lineage tracing, organoid formation assays, and transcriptomic analyses to characterize the contribution of ALDH1A1^+^ stem cells in the endometrium.

## Results

### Mouse ALDH^HI^ cells have a higher organoid formation capacity and stem-like signatures than ALDH^LO^ cells

**Figure 1.**
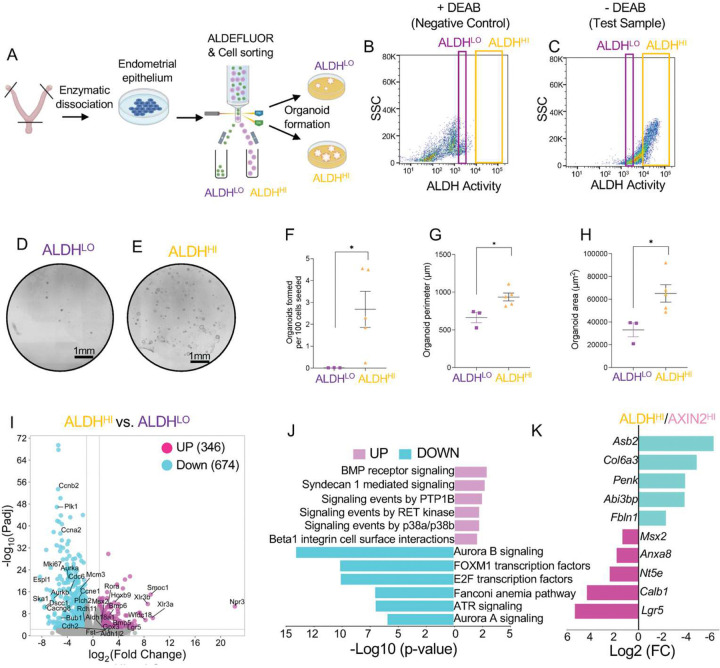
Mouse endometrial epithelial ALDH^HI^ cells have higher organoid formation capacity and stemness transcriptomes. A-C) Strategy to isolate and establish ALDH^HI^ and ALDH^LO^ cells from adult WT mouse epithelial organoids using the ALDEFLUOR assay. D-E) Organoid formation assay performed immediately after luminal epithelial cell isolation by plating equal numbers of viable ALDH^LO^ (D) and ALDH^HI^ (E) epithelial cells. ALDH^LO^ and ALDH^HI^ organoids were cultured for two weeks and passaged once prior to the organoid formation assays and transcriptomic analyses. F-H) Organoid formation rate (F), organoid perimeter (G) and area (H) were assessed by quantifying the total number of organoids formed per 100 cells seeded. Assays were performed using the cells pooled from 7–9 WT adult mice at estrus three independent times. I-J) Transcriptomic profiling of ALDH^HI^ vs. ALDH^LO^ mouse organoids was performed, and the total number of differentially expressed genes (DEG) was determined (I). The total number of up- and down-regulated genes was displayed as a volcano plot. J) Gene ontology analysis was performed on the up- and down-regulated genes between the ALDH^LO^ vs. ALDH^HI^ organoids. K) Comparison of total genes that are conserved as up- or down-regulated between ALDH^HI^/AXIN2^HI^ and ALDH^LO^/AXIN2^LO^ cell, selected genes involved in stemness signatures are displayed. Graphs show mean ± SEM and analyzed using a non-parametric Mann-Whitney Test, *, p<0.05; **, P<0.01; ***, p<0.001.

To define the stem-like characteristics of mouse endometrial epithelial cells with high aldehyde dehydrogenase activity, we used the ALDEFLUOR assay (Storms et al., 1999), which separates cells by their ability to metabolize a BODIPY-labeled ALDH substrate (Figure 1A–B). We collected adult female WT mice during the estrus phase and dissociated their endometrial epithelial cells to sort by FACS into ALDH^HI^ and ALDH^LO^ populations using the ALDEFLUOR assay (Figure 1A–C). Because organoid formation ability is indicative of stemness, we expanded the cells and subjected ALDH^HI^ and ALDH^LO^ cells to organoid formation assays and transcriptomic profiling by RNAseq. Compared to ALDH^LO^ organoids, ALDH^HI^ organoids had a higher organoid formation rate (0.01% vs. 2.69%, n=3 and n=5, p<0.05), perimeter (664 ±119 vs. 936 ±117 μm, p<0.05) and area (33,017 ±10,580, vs. 65,068 ±17,014 μm^2^, p<0.05) (Figure 1D–H).

Using RNAseq we identified that 346 transcripts were increased and 674 transcripts were decreased (>1, <−1 log2FC, Adj. p-value <0.005) in ALDH^HI^ vs. ALDH^LO^ organoids (Figure 1I). Gene ontology analysis of the DE genes showed that compared to ALDH^LO^ cells, the ALDH^HI^ cells had decreased expression of genes involved in ‘Aurora B signaling’ and ‘E2F transcription factor network,’ with genes such as *Ccnb2, Ccna2, Ccne1, Aurka, Mki67,* and *Bub1*, being significantly decreased in ALDH^HI^ vs. ALDH^LO^ cells (Figure 1I–J, **Supplemental Figure 1A, Supplemental Table S1**). Genes involved in categories related to stem cell activity, such as ‘BMP receptor signaling’, were increased in the ALDH^HI^ vs. ALDH^LO^ cells, with genes such as *Bmp4, Bmp5, Bmp6, Fst, Cdh2, Lgr5, Wnt9a, and Fzd3* showing higher expression. To identify similarities with other stemness cells in the mouse endometrium, we compared the DE genes in the ALDH^HI^ vs. ALDH^LO^ list with the DE genes from the Axin2^HI^ vs. Axin2^LO^ mouse endometrial epithelial cells (Syed et al., 2020). We found that 21 genes were increased, and 19 genes were decreased in both ALDH^HI^ and AXIN2^HI^ cell populations (**Supplemental Figure 1B, Supplemental Table S1**). We determined that the overlap between ALDH^HI^ and Axin2^+^ stemness marker genes was significantly greater than expected by chance for both upregulated (21/346 genes, 1.81-fold enrichment, p = 0.0067) and downregulated (19/674 genes, 1.67-fold enrichment, p = 0.021) gene sets (hypergeometric test, universe = 23,182 genes). Specifically, *Lgr5, Calb1,* and *Msx2* were all identified as increased in both cell types (Figure 1K).

Keratin genes encode intermediate filament proteins that are used to classify epithelial subtypes (*i.e.,* basal vs. simple columnar epithelium) but can also indicate progenitor state, differentiation status, or wound-healing response (Cohen et al., 2022). We found differences in keratin gene expression between ALDH^HI^ and ALDH^LO^ cells (**Supplemental Figure 1C, Supplemental Table S1**), with ALDH^HI^ cells showing higher expression of *Krt17,* which is associated with cell differentiation, wound healing responses, and localizes with *SOX9*^+^/*LGR5*^+^ progenitors in the human endometrium (Cohen et al., 2024; Garcia-Alonso et al., 2021). ALDH^LO^ cells had higher expression of several other keratin-related genes (*i.e., Krt15, Krt4, Krt12*) when compared to ALDH^HI^ cells, suggesting unique differentiation potential between the two cell types. Because of the critical roles of the steroid hormones on epithelial cell proliferation, we also analyzed the expression levels of the estrogen (*Esr1*) and progesterone receptors (*Pgr*) and observed comparable levels between ALDH^LO^ and ALDH^HI^ cells (**Supplemental Table S1**). Overall, these studies show that ALDH^HI^ cells display enhanced clonogenic and regenerative capacity in organoid assays, consistent with an adult stem cell state.

### Endometrial Aldh1a1^+^ cells display gene expression signatures consistent with a stem cell state

**Figure 2.**
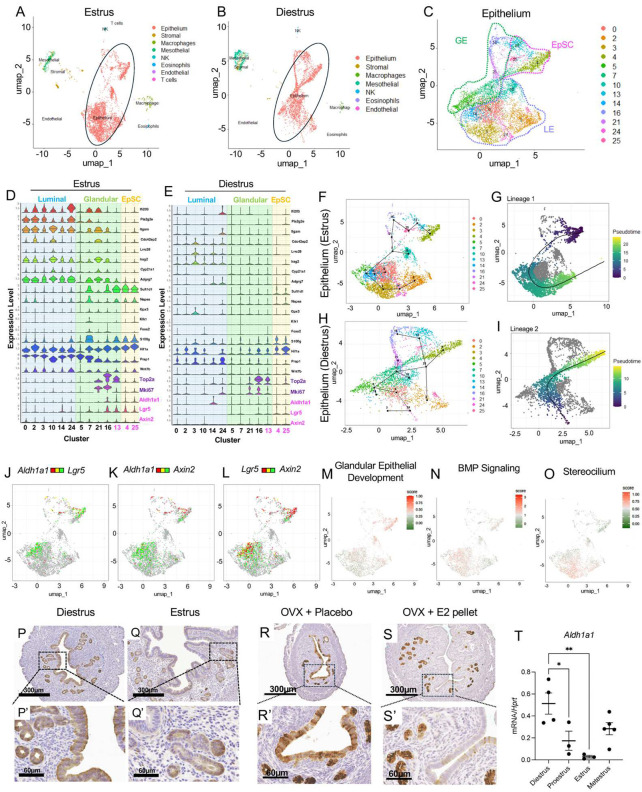
ALDH1A1 is expressed in cells with epithelial stem cell characteristics. A-B) UMAP displaying the various cell types identified by scRNAseq of enriched endometrial epithelium from WT 6-week-old mice in the estrus (A) and diestrus (B) phases. Epithelial cells were sub-clustered to identity different cell identities (C) and classified into luminal, glandular, and epithelial stem cell (EpSCs) based on the expression of key markers (D, estrus; E, diestrus). F-I) Pseudotime analysis of the epithelial cell types in estrus (F, G) and diestrus (H,I) was performed to identify the trajectory of differentiation, which shows that EpSCs give rise to glandular and epithelial cell lineages. J-L) Dual feature plots showing the overlapping and unique expression patterns of *Aldh1a1/Lgr5* (J), *Aldh1a1/Axin2* (K), and *Lgr5/Axin2* (L) in the epithelial cell clusters in estrus. M-O) A signature score was assigned to the epithelial cells from the estrus phase to determine how strongly genes involved in ‘Glandular Epithelial Development’ (M), ‘BMP signaling’ (N), and ‘Stereocilium’ (O) are expressed. P-S) ALDH1A1 immunohistochemistry in the uterus of adult WT mice during the diestrus (P-P’) and estrus (Q-Q’) phases, or in WT ovariectomized mice without (R-R’) or with an E2 pellet for 90 days (S-S’). T) *Aldh1a1* was also quantified in the uterus of 6–8-week-old WT mice collected at different times during the estrous cycle. Experiments were repeated in more than three mice per group. Data in T are displayed as mean ± SEM analyzed by a One-Way ANOVA test with a Tukey’s post-hoc test. *, p<0.05; **, P<0.01; ***, p<0.001. *UMAP*, uniform manifold approximation and projection; *EpSC*, epithelial stem cell; *BMP*, bone morphogenetic protein.

Previous studies used scRNAseq analyses of postnatal uteri to identify a niche of potential endometrial stem cells that repopulate the regenerating endometrium throughout life (Spencer et al., 2023; Wu et al., 2017). Other studies have analyzed endometrial epithelial cells of adult cycling mice but have captured only a few epithelial cells, preventing in-depth analyses of cells with stem/progenitor signatures (Winkler et al., 2024). We enriched endometrial epithelial cells from adult cycling wild-type (WT) mice and analyzed the expression of approximately 5,984 total cells from mice during the estrus phase and 3,995 cells from mice in diestrus phase using scRNAseq on the 10X Genomics platform (Figure 2). We obtained between 55,000–98,000 reads per cell and detected 18,000–24,000 genes per cell. Cells were clustered on Seurat version 5.2.0 using uniform manifold approximation and projection (UMAP). We then classified cell types by identifying differentially expressed (DE) genes between the clusters and using markers that were previously described (Wang et al., 2023; Wang et al., 2020; Winkler et al., 2024). This classification method identified eight different cell types (epithelial, stromal, macrophages, mesothelial, natural killer cells (NK), eosinophils, endothelial, and T-cells) (Figure 2A–B) within our samples. Of these cells, approximately 7,569 (4,488 from estrus, 3,081 from diestrus) were classified as epithelial cells, indicating that we effectively enriched the epithelial cell population.

We performed further analyses in the epithelial cells by sub-clustering them with previously annotated markers of the luminal (*Ifi203, Pla2g2e, Itgam, Cdc42ep2, Lrrc26, Irag2, Cyp21a1, Adrg7)* and glandular epithelium (*Sult1d1, Napsa, Gpx3, Klk1, Foxa2*) (Padilla-Banks et al., 2023) (Figure 2C, **Supplemental Figure S2D-E**). Clusters 5, 7, 21 and 16 were classified as glandular epithelial cells, and clusters 0, 2, 3, 10, 14, and 24 were classified as luminal epithelial cells (Figure 2C). To identify whether a niche of epithelial stem cells (EpSC) was represented in each phase, we analyzed the expression of *Aldh1a1, Axin2, Lgr5, Gstm7, En2,* and *Wnt7a* in the epithelial cells and found that *Aldh1a1, Axin2* and *Lgr5* were enriched in clusters 4, 25, and 13 (Figure 2D–E, **Supplemental Figure S2A-B**). Co-expression of *Aldh1a1, Lgr5,* and *Axin2* in these cells could be observed in the dual feature plots of epithelial cell subclusters from mice in estrus (Figure 2J–L). EpSCs present in clusters 4 and 25 had lower expression of proliferation markers *Top2a* and *Mki67* relative to other clusters, suggesting that they were in a quiescent state (Figure 2D–E, **Supplemental Table S2**). In addition to expressing *Aldh1a1, Axin2,* and *Lgr5,* cluster 13 also expressed *Top2a* and *Mki67,* indicating that these cells are less quiescent (Figure 2D–E). Other notable differences included expression of *Aldh1a1* in the luminal cell cluster 14 in the diestrus phase epithelium (Figure 2E), a shift that we also observed by ALDH1A1 IHC (Figure 2P–Q’).

To further characterize the transcriptomes of quiescent EpSCs, we analyzed differentially expressed (DE) genes in clusters 4 and 25 versus the other epithelial cells in our dataset (**Supplemental Table S2**), which showed that approximately 170 conserved genes were increased (>1 log2FC, Adj. p-value <0.01) and 52 were decreased (<−1 log2FC, Adj. p-value, 0.01) in the EpSC of mice analyzed during both the estrus and diestrus phases. Differentially expressed genes included *Calb1, Lpar3, Cited4, and Tgfbi*, whose expression increases in the epithelium during postnatal uterine maturation (Spencer et al., 2023). *Susd2,* a marker of endometrial stem cells (Masuda et al., 2012), was also increased in the EpSC clusters, along with *Cyp26a1,* a member of retinoic acid signaling (Isoherranen and Zhong, 2019), and *Tgfbi,* which is induced by the transforming growth factor beta signaling pathway, stimulates NOTCH signaling, and maintains glioma stem cell identity (Chen et al., 2024; Corona and Blobe, 2021; Lee et al., 2023). Comparison with a previously published scRNAseq dataset (Padilla-Banks et al., 2023) also showed that clusters 4 and 25 shared transcriptomic signatures with the EpSC cluster identified in their analysis (**Supplemental Figure S2C**).

We then determined the dynamic continuum of the EpSCs using trajectory analysis of the epithelial subclusters using Slingshot pseudotime analysis (Figure 2F–I, **Supplemental Figure S2F-L**). This analysis showed that the EpSC clusters from the estrus phase transitioned from the quiescent *Aldh1a1, Lgr5, Axin2* expressing cells in clusters 4 and 25 toward the proliferative *Aldh1a1, Lgr5, Axin2*-expressing cluster 13, which expressed the proliferative *Mki67* and *Top2a* markers (Figure 2F–G). The trajectory then progressed toward the glandular epithelial cells and ended with the cells in the luminal epithelium (Figure 2F–G), with additional projected trajectories shown in **Supplemental Figure S2F-G**. The trajectory for epithelial cells in the diestrus phase had more branching points than those of the estrus phase cells (Figure 2H–I, **Supplemental Figure S2H-L**), suggesting that *Aldh1a1*-expressing cells are controlled by the dynamic levels of sex hormones during the estrous cycle (Nilsson et al., 2015). Accordingly, gene enrichment analysis of the epithelial clusters showed that cells in the EpSC clusters displayed high signature scores for the categories of ‘Glandular Epithelial Development’ and ‘BMP Signaling,’ while luminal cells had higher scores of genes enriched in ‘Stereocilium’ categories (Figure 2M–O). Our analyses of adult cycling endometrial epithelium suggest that ALDH1A1^+^ cells are enriched in cell types with stem cell signatures.

### Expression and localization of ALDH1A1 is controlled by estrogen and progesterone in the adult cycling uterus

We validated the dynamic localization of *Aldh1a1*-expressing cells identified by scRNAseq using IHC in the WT uterus of adult mice in estrus and diestrus (Figure 2P–T). Expression of ALDH1A1 was detected in both the luminal and glandular epithelium during diestrus (Figure 2P–P’) but localized to the glandular crypts during the estrus phase (Figure 2Q–Q’). We also observed this hormone-dependent expression of *Aldh1a1* at the mRNA level, where *Aldh1a1* was highest during the diestrus phase compared to the pro-estrus and estrus phases (Figure 2T). Ovariectomy led to diffusing ALDH1A1 expression throughout the luminal and glandular epithelium, while a long-term E2 treatment restricted ALDH1A1 expression to the glandular crypts (Figure 2R–S’). These results confirmed that the localization of ALDH1A1-expressing cells is controlled by steroid hormones in the adult uterus.

To further investigate the hormone-dependent expression patterns of ALDH1A1, we treated ovariectomized mice with a series of hormones (**Figure S3A**). Ovariectomy, and thus depletion of endogenous estrogen (E2) and progesterone (P4), led to expression of ALDH1A1 in both the luminal and glandular epithelium (**Figure S3B-B’)**. In contrast, treatment with P4, E2, or a combination of P4+E2, increased localization toward the glandular crypts (**Figure S3C-E’**). Treatment with P4 alone decreased the gene expression levels of *Aldh1a1* when compared to uterine tissues from ovariectomized or E2-treated mice (**Figure S3F**). Thus, in the absence of hormones, ALDH1A1 was diffusely expressed throughout the luminal and glandular epithelium. On the other hand, E2 and P4 directly impacted the localization of ALDH1A1^+^ cells in the epithelium.

### Lineage tracing of Aldh1a1^+^ cells in the postnatal period reveals they are long-lived cells of the endometrium

**Figure 3.**
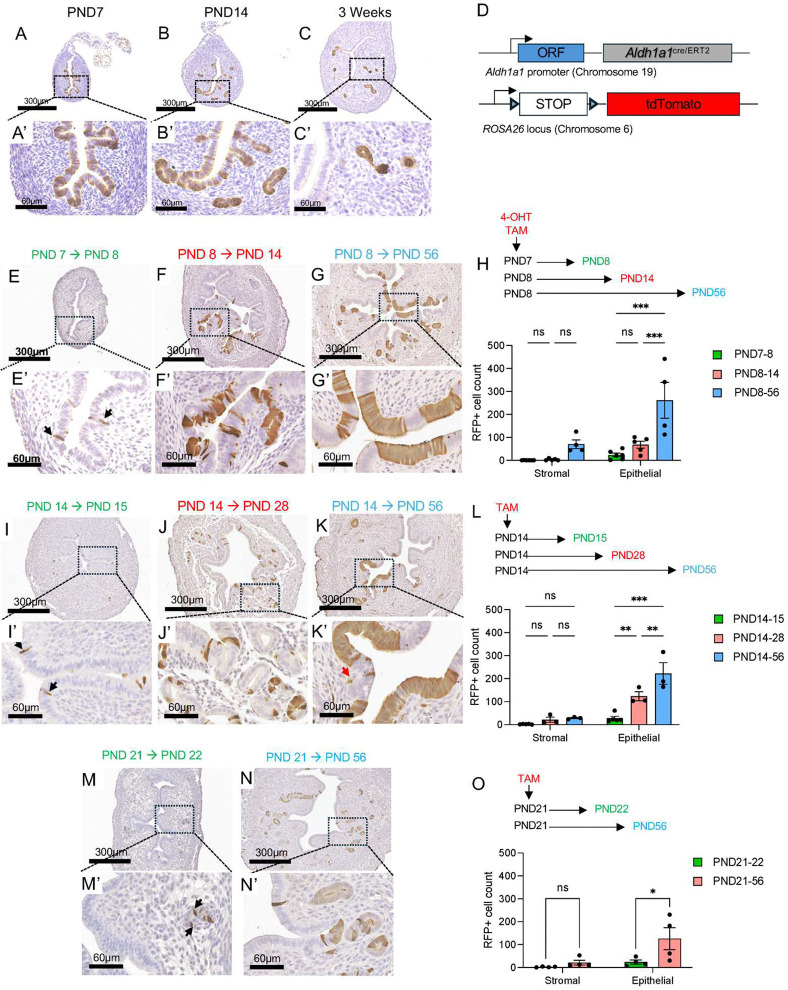
Lineage tracing reveals the contribution of ALDH1A1^+^ cells to endometrial integrity. A-C’) ALDH1A1 immunohistochemistry in the WT mouse uterus at PND7 (A-A’), PND14 (B-B’), and 3-weeks (C-C’). D) Schematic of the tamoxifen-inducible ALDH1A1 reporter allele and experimental scheme used for lineage tracing in the postnatal endometrium. E-N’) RFP immunohistochemistry was used to detect the ALDH1A1-tdTomato-expressing cells in the uterus when tracing was performed from PND7 to PND8 (E-E’), PND8 to PND14 (F-F’), PND8 to PND56 (G-G’), PND14 to PND15 (I-I’), PND14 to PND28 (J-J’), PND14 to PND56 (K-K’) or PND21 to PND22 (M-M’) or PND21 to PND56 (N-N’) with tamoxifen (0.15 mg/g body weight). H, L, O) Quantification of RFP^+^ cells was performed and are presented as total RFP^+^ endometrial epithelial or stromal cells. Black arrows (E’, I’, M’) indicate singly labeled RFP^+^ cells, red arrow (K’) indicates RFP^+^ stromal cell. Images represent staining that was performed in 3 or more mice per timepoint. Data are presented as mean ± SEM analyzed by a Two-Way ANOVA with a Sidak test for multiple comparisons. *, p<0.05; **, P<0.01; ***, p<0.001. *PND,* postnatal day; *TAM*, tamoxifen; *ORF*, open reading frame.

ALDH1A1 has a dynamic expression pattern in the postnatal endometrium, displaying prominent expression throughout the epithelium at postnatal day 7 (PND7) (Figure 3A–A’), gradual accumulation in the endometrial glands as their development progresses at PND14 (Figure 3B–B’), and restriction to the glandular crypts of more mature glands by PND21 (Figure 3C–C’). This expression pattern resembles WNT-related signaling molecules, such as AXIN2 and LGR5, which are previously characterized drivers of endometrial regeneration (Seishima et al., 2019; Syed et al., 2020). To define the long-term contributions of ALDH1A1^+^ cells in the endometrium, we generated an ALDH1A1^tdTomato^ reporter mouse by crossing an *Aldh1a1*^cre/ERT2/+^ mouse to a *ROSA26*^tdTomato/TdTomato^ cre-reporter line, which would label ALDH1A1^+^ cells at the time of induction and their subsequent progeny with red fluorescent protein (RFP) (Figure 3D) (Madisen et al., 2010; Poulin et al., 2018).

Singly labeled cells were obtained by using a low-dose of 4-OHT (0.5μg/g) and verified following 1 day of tracing (PND7→ PND8) (Figure 3E–E’). Increasing numbers of luminal and glandular cells were detected 6 days later (PND8→ PND14) (Figure 3F–F’) with increased expansion in both the glandular and luminal epithelium following long-term labeling (PND8 → PND56) (Figure 3G–G’). This gradual increase of RFP-labeled epithelial cells was quantified, showing that ALDH1A1^+^ cells expanded and remained as long-term resident cells of the luminal and glandular endometrial epithelium (Figure 3H). When tracing was begun at PND14, a time when endometrial glands begin to invaginate into the underlying stroma (Figure 3I–L), singly labeled RFP^+^ cells were detected in luminal and glandular epithelium following a short-term trace (PND14 → PND15, Figure 3I–I’). Increasing numbers of cells were observed in the mice traced from PND14 → PND28 and PND14 → PND56, showing that at this timepoint, ALDH1A1^+^ cells contribute to both luminal and glandular cells of the endometrium (Figure 3J–J’, K–K’, L). When ALDH1A1^+^ cells were labeled at PND21, single glandular epithelial cells were RFP^+^ (Figure 3M–M’), and these were detected in larger patches of glandular and luminal epithelial cells when traced from PND21 → PND56 (Figure 3N–N’, O). Total RFP^+^ cells per visual field across both the stromal and epithelial compartments showed that stromal cells were labeled at each time point we analyzed with no significant increase in ALDH1A1^+^ stromal cells over time (Figure 3H,L,O). Thus, our lineage tracing studies indicate that ALDH1A1^+^cells can be detected in glandular, luminal, and stromal compartments within the endometrium during short and long labeling periods.

### ALDH1A1^+^ cells are dynamically expressed in the adult endometrium of cycling mice

**Figure 4.**
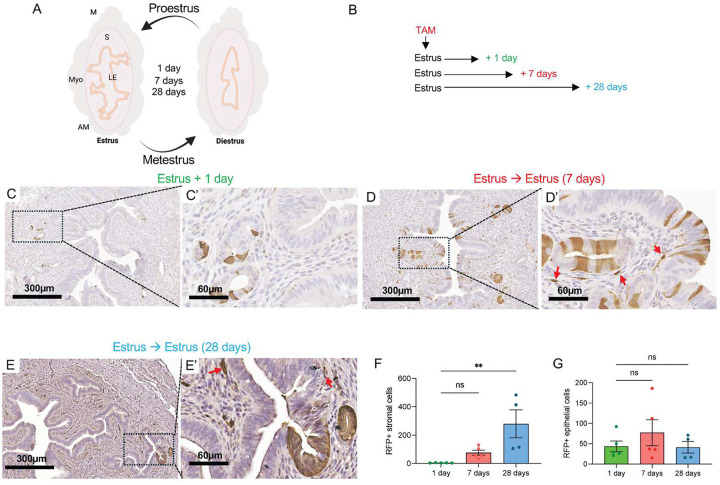
Tracing of ALDH1A1^+^ cells in the adult cycling uterus identified positive cells in the epithelium and stroma. A) Schematic diagram demonstrating the reabsorption and reorganization that occurs between estrous phases in the adult mouse uterus. Uterine structures are labelled as: M for mesometrial, AM for anti-mesometrial, Myo for myometrium, S for stromal compartment, and LE for luminal epithelium. B) Timeline for tamoxifen-dependent tomato labeling in ALDH1A1-expressing cells in the adult mice when traced for one day, seven days, or 28 days after Tamoxifen administration. C-E’) RFP immunohistochemistry was used to detect labeled cells when tracing was performed in the adult mouse for 1 day (C-C’), seven days (D-D’), or 28 days (E-E’). Red arrows indicate detection of RFP in the sub-epithelial stromal cells. F-G) Quantification of RFP^+^ cells in the epithelial and stromal compartments one day, seven days, or four days after tamoxifen administration. Data are presented as mean ± SEM analyzed by a Two-Way ANOVA with a Sidak test for multiple comparisons. *, p<0.05; **, P<0.01; ***, p<0.001. *TAM*, tamoxifen.

To determine the fate of ALDH1A1^+^ cells in the adult cycling endometrium, we began tracing ALDH1A1^+^ cells during the estrus phase of their cycle (Figure 4A–B), a time when ALDH1A1 cells were enriched in the glandular crypts (Figure 2Q). We verified the number of epithelial and stromal cells that were labeled in the endometrium following one day of tracing (Figure 4C, F–G). After 7 and 28 days of tracing, several glandular, luminal and sub-epithelial stromal cells were labeled (Figure 4D–E’). When tracing was carried out for 28 days, the number of stromal but not epithelial labeled cells expanded relative to one day of tracing (Figure 4F–G). Hence, when lineage tracing studies were performed in adult mice, expansion of ALDH1A1^+^ stromal cells was more readily observed than expansion of ALDH1A1^+^ epithelial cells. This suggested that ALDH1A1^+^ cells play a critical role in stromal cell expansion in the adult endometrium.

### ALDH1A1^+^ cells are detected in the postpartum endometrium

**Figure 5.**
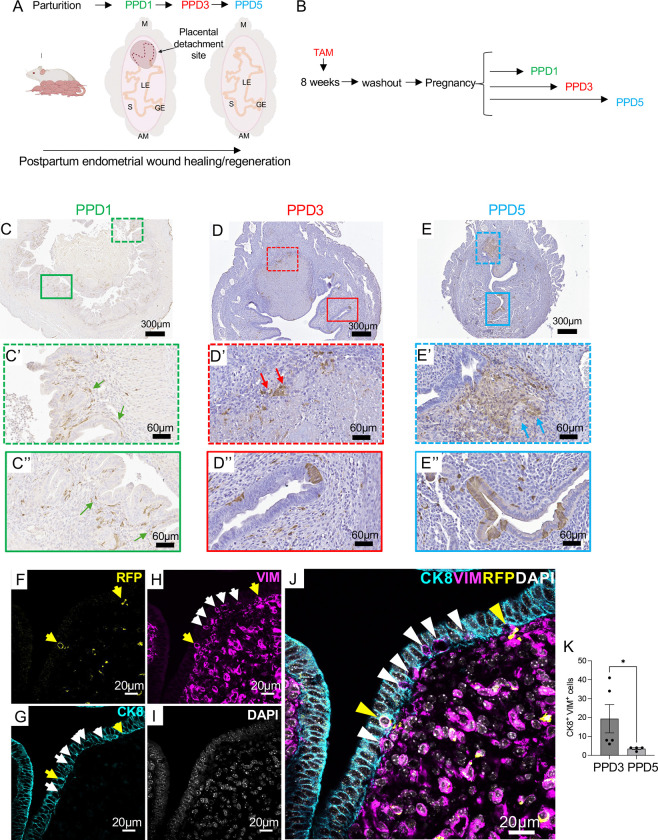
ALDH1A1^+^ cells are detected in the post-partum regenerating endometrium. A) Schematic highlighting post-partum repair occurring at PPD1, PPD3, and PPD5. Uterine structures are labeled as: M for mesometrial, AM for anti-mesometrial, S for stromal compartment, LE for luminal epithelium, and GE for glandular epithelium. B) ALDH1A1 lineage tracing was begun in the *Aldh1a1*^cre/ERT2^;*ROSA26*^tdTomato/tdTomato^ mice at 2 months of age. Two months after TAM administration, the mice were mated, and their uteri were collected at PPD1, PPD3, and PPD5. C-E”) RFP immunohistochemistry shows the presence of ALDH1A1^+^ cells in areas near the placental detachment sites collected at PPD1 (C-C”), PPD3 (D-D”), and PPD5 (E-E”). Arrows indicate the RFP^+^ subepithelial stromal cells (C’-C”) and RFP^+^ stromal cells near the placental detachment sites (D’, E’). F-J) Immunofluorescence staining of uterine cross-sections with cytokeratin 8 (CK8, cyan), red fluorescence protein (RFP, yellow), vimentin (VIM, magenta), and DAPI (white). Yellow arrowheads (F, G-J) show the presence of cells that are CK8^+^/RFP^+^/VIM^+^; White arrowheads indicate cells that are CK8^+^/VIM^+^. Images represent groups of more than three animals analyzed per group. K) Analysis of the cells expressing CK8^+^/VIM^+^ in the uterine cross-sections at PPD3 and PPD5. Data are presented as mean ± SEM of positive cells per imaged field and analyzed by a Mann-Whitney test (M). *, p<0.0033; **, P<0.002; ***, p<0.001. *TAM*, tamoxifen; *PPD,* post-partum day.

We traced the fate of ALDH1A1^+^ cells in the postpartum endometrium to observe their fate during endometrial regeneration (Figure 5). We administered tamoxifen to mice to initiate tracing two months prior to mating and then collected their uterine tissues 1, 3 and 5 days postpartum (PPD1, PPD3 and PPD5) (Figure 5A–B). By analyzing the uterine tissues near the placental detachment site, we observed that RFP positive cells were prominent in the endometrial stromal cells that were adjacent to the luminal epithelium (Figure 5C–C’, *green arrows*). RFP^+^ cells were also observed in the stromal cells near the placental detachment sites at PPD1 and PPD3 (Figure 5D’–E’, red & blue arrows) and in limited luminal epithelial cells (Figure 5D”,E”). Quantification of RFP^+^ cells throughout these postpartum phases showed more frequent ALDH1A1^+^ stromal cells (360 ± 103, PPD1, n=3; 217 ± 107, PPD3, n=3; 254 ± 32, PPD5, n=4) than ALDH1A1^+^ epithelial cells in the regenerating endometrium (65 ± 65, PPD1, n=3; 20 ± 10, PPD3, n=3; 114.25 ± 39, PPD5, n=4) (**Figure S4**). When we performed immunofluorescence imaging of CK8 (epithelial cell marker), VIM (stromal cell marker), and RFP, we observed that the total number of VIM^+^CK8^+^ transitional stromal/epithelial cells was significantly higher in the PPD3 endometrium when compared to PPD5 (19.4 ± 7.48 vs. 3.5 ± 0.5, p<0.05), suggesting the presence of transitional VIM^+^CK8^+^ cells is abundant at PPD3, with a subset of these transitional cells also being RFP^+^ (Figure 5F–K). Our results showed that ALDH1A1^+^ cells were present in the regenerating endometrium, with some also displaying expression of transitional CK8^+^ and VIM^+^ cell markers.

### Ablation of ALDH1A1^+^ cells disrupts endometrial epithelial expansion in vitro and in vivo

**Figure 6.**
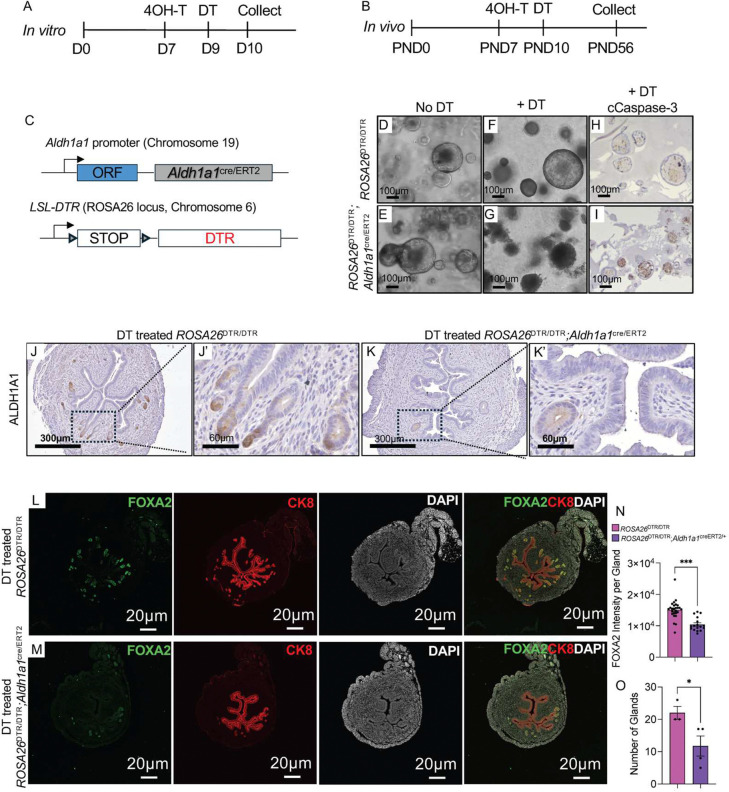
Ablation of ALDH1A1^+^ cells impairs organoid expansion and reduces endometrial glands in adult mice. A-C) Diphtheria toxin-mediated ablation of ALDH1A1+ cells was obtained *in vitro* (A) and *in vivo* (B) by crossing *Aldh1a1*^cre/ERT2^;*ROSA26*^tdTomato/tdTomato^ mice to a line containing a conditional diphtheria toxin receptor (DTR). D-I) Organoids from adult control (DTR^f/f^) and experimental (*Aldh1a1*^cre/ERT2^;*DTR*^*f/f*^) mice were established and expanded in culture for two passages. Once established (D-E), the organoids were treated with DT (F-G) and visualized, fixed and stained with cleaved caspase-3 antibody (H-I). J-K) The impact of DT-mediated ablation of ALDH1A1+ cells was determined in mice treated with TAM at PND7, and with DT at PND10. Uterine tissues were collected and analyzed at PND56 using immunohistochemistry to detect ALDH1A1 (J-K’). **L-M**) Glands were visualized in the control mice (**L**) and experimental (M) by staining with FOXA2 (green), cytokeratin 8 (red), and DAPI (white) using confocal imaging. **N**-O) FOXA2 intensity per gland (N) and glandular number (O) were quantified in the uterine cross-sections of >3 mice per genotype. The results are displayed as mean ± SEM and analyzed using a two-tailed t-test, *, p<0.05; **, P<0.01; ***, p<0.001.

To investigate how ablation of the ALDH1A1^+^ cell population affects epithelial expansion, we ablated ALDH1A1^+^ cells *in vitro* and *in vivo* using diphtheria toxin (DT)-mediated ablation by crossing *ROSA26*^DTR/DTR^ mice harboring conditionally expressed diphtheria toxin receptors (DTR) to *Aldh1a1*^cre/ERT2/+^ mice (Figure 6A–C). We then established endometrial epithelial organoids from adult female *ROSA26*^DTR/DTR^;*Aldh1a1*^cre/ERT2/+^ mice. Once mature organoids were obtained, Cre activity was induced by treating them with 4-OHT for two days followed by DT treatment (Figure 6A). Organoids from the control (*ROSA26*^DTR/DTR^) and experimental mice (*ROSA26*^DTR/DTR^;*Aldh1a1*^cre/ERT2/+^) were intact under phase contrast microscopy prior to DT treatment (Figure 6D–E). After DT treatment, the organoids from the control *ROSA26*^DTR/DTR^ mice were intact, while those from the experimental group, *ROSA26*^DTR/DTR^;*Aldh1a1*^cre/ERT2/+^, began to disintegrate, appeared dark, and were positive for cleaved caspase-3 (Figure 6F–I).

To examine the effects of ALDH1A1 ablation *in vivo,* we treated control and experimental mice with tamoxifen at PND7 to induce Cre activity in the ALDH1A1^+^ cells, followed by treatment with DT at PND10. When we analyzed the mice at P56, we observed decreased ALDH1A1 expression in the experimental mice (Figure 6J–K’). We then immunostained uterine cross-sections with CK8 and FOXA2, which showed that the experimental mice had fewer glands compared to controls (11.75 ± 8.2 vs. 22 ± 3.4, p<0.05) and decreased FOXA2 intensity per gland (10,434 ± 2,136 vs. 15,079 ± 3,006, p<0.001) relative to the controls (Figure 6L–O). We determined that the decreased in FOXA2^+^ cells in the experimental mice was not the result of non-specific DT-mediated cell death, as similar levels of cleaved caspase 3-positive cells were detected in the DT-treated control *ROSA26*^DTR/DTR^ and *ROSA26*^DTR/DTR^;*Aldh1a1*^cre/ERT2/+^ mice 4 days post-diphtheria toxin administration (**Figure S3G-H’**). These results indicated that the ablation of ALDH1A1 cells decreases epithelial organoid expansion *in vitro* and FOXA2 expression *in vivo*.

### ALDH^HI^ epithelial cells of the human endometrium display properties and signatures of adult stem cells

**Figure 7.**
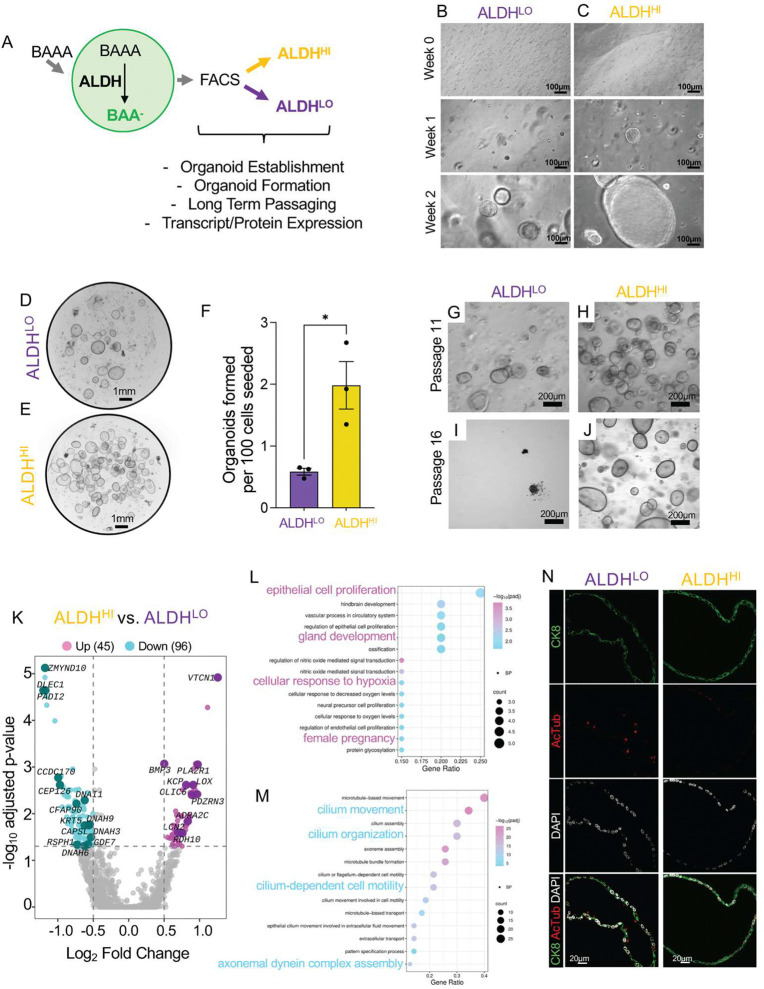
ALDH^HI^ cells from eutopic endometrium display organoid formation capacity and different transcriptomic signatures than ALDH^LO^ cells. A) Schematic approach to isolate ALDH^HI^ and ALDH^LO^ cells from human endometrial epithelial organoids with the ALDEFLUOR assay. B-C) Organoid establishment was assessed in freshly isolated ALDH^LO^ and ALDH^HI^ epithelial cells plated at equal densities. D-F) Organoid formation assay was performed by plating equal numbers of viable ALDH^LO^ (D) and ALDH^HI^ (E) cells followed by quantifying the total number of organoids that were established per 100 cells plated (F). Graph displays the mean ± SEM of organoids from one patient in technical replicates and analyzed using a two-tailed t test, *, p<0.05; **, P<0.01; ***, p<0.001. G-J) Images comparing the growth of ALDH^LO^ (G,I) and ALDH^HI^ (H,J) organoids at passage 11 (G-H) or passage 16 (I,J) from one patient. K) Volcano plot showing the total number of differentially expressed transcripts in the ALDH^HI^ vs ALDH^LO^ eutopic organoids from three different patients. L-M) Gene enrichment analysis of increased (L) and decreased (M) genes in ALDH^HI^ eutopic vs ALDH^LO^ eutopic organoids. N) Immunostaining of eutopic ALDH^LO^ and ALDH^HI^ organoids stained with cytokeratin 8 (CK8, green), Acetylated-α-tubulin (AcTub, red), and DAPI (white).

Previous studies showed that ALDH1A1 is enriched in the glandular epithelial cells in the basalis human endometrium, a site considered to be enriched with adult stem cells (Ma et al., 2020). To determine the regenerative potential of ALDH^HI^ cells in the human endometrium, we established organoids from the eutopic endometrium of donors. We separated ALDH^HI^ and ALDH^LO^ cells using the ALDEFLUOR assay and expanded them *in vitro* (Figure 7A). Organoid formation assays identified that ALDH^HI^ cells had a higher capacity to form organoids than ALDH^LO^ cells (1.98 ± 0.38 vs 0.58 ± 0.055, p=0.022; 9.04 ± 0.72 vs 1.7 ± 0.38, p=0.0008) (Figure 7B–F, **Supplemental Figure S5A-C,** main figures show representative results from one patient sample performed in technical replicates, with additional patient samples included in the supplement). ALDH^HI^ cells were also maintained in culture over longer passages, though the difference was not statistically significant (23 ± 3 vs. 14 ± 2 passages, p=0.0576, n=3 donors) (Figure 7G–J).

To determine the gene expression differences between the two cell types, we performed transcriptomic profiling in the ALDH^HI^ and ALDH^LO^ organoids from the eutopic endometrium of three donors. Analyses of the differentially expressed genes showed that 45 genes were increased (>0.5 log2FC, Adj. p-value <0.05) and 96 genes were decreased (<−0.5 log2FC, Adj. p-value <0.05) in ALDH^HI^ vs. ALDH^LO^ organoids (Figure 7K, **Supplemental Table S3**). Gene ontology analyses showed that ALDH^LO^ cells had higher expression of genes enriched in ciliated cells, such as *RSPH4A, CFAP73, DNAI1, SPAG17,* and several others, while ALDH^HI^ had higher expression of genes involved in epithelial cell proliferation and gland development, such as *GATA2,VEGFA* and *IGFBP3* (Figure 7K–M, **Supplemental Figure S6, and Supplemental Table S3**). Genes that were increased in the ALDH^HI^ cells also included genes involved in stemness, such as *BMP3, ADH1C, KCP,* and *PLA2R1* (Figure 7K, **Supplemental Table S3**). We did not identify any differences in the expression of *ESR1, PGR,* or other nuclear hormones between groups (**Supplemental Table S3**). Immunostaining of ALDH^HI^ and ALDH^LO^ organoids from eutopic organoids confirmed that significantly more ciliated cells were present in ALDH^LO^ organoids when compared to ALDH^HI^ eutopic organoids (7.2 ± 1.56 vs. 0.77 ± 0.42, p<0.001) (Figure 7N). Thus, in human endometrial epithelial cells, ALDH^HI^ cells displayed a higher organoid formation rate, expressed fewer ciliated cell-associated genes, and had increased levels of stemness genes, consistent with characteristics of adult stem cells of the human basalis endometrium.

## Discussion

Key studies have identified and characterized endometrial stem cells by analyzing them in the endometrial tissues of postmenopausal women, a tissue that is enriched in basalis cells. Through studies of the postmenopausal basalis, *AXIN2, SOX9, SSEA1,* and *CDH2*-positive cells are now widely accepted as epithelial cell progenitors localized in the human basalis endometrium (Nguyen et al., 2012) (Nguyen et al., 2017; Valentijn et al., 2013). Additionally, *SUSD2*^+^ stromal cells located in the perivascular areas possess more colony formation capacity than *SUSD2*^−^ cells. This population of cells also expresses CD140b (PDGFRβ) and CD146, which are considered to be endometrial mesenchymal stem-like cells (Masuda et al., 2012). Due to its enriched location in the basalis endometrium and colocalization with CDH2, ALDH1A1 has also been proposed as a stem cell marker in the endometrium (Ma et al., 2020).

Recent scRNAseq and spatial transcriptomic analyses of human endometrium have confirmed many of the proposed endometrial stem cell markers, with *SOX9*^+^ cells enriched in the basal endometrium which give rise to *SOX9*^+^/*LGR5*^+^ cells (Garcia-Alonso et al., 2021; Wang et al., 2020). WNT and NOTCH were also identified as critical growth factors controlling endometrial stem cell differentiation, with WNT activator signals controlling luminal epithelial cell development, and NOTCH maintaining stemness in the basalis (Garcia-Alonso et al., 2021). In a high-resolution single cell reference atlas of the human endometrium, a population of *CDH2*^+^*/SOX9*^+^*/AXIN2*^+^*/ALDH1A1*^+^ cells was identified in the basalis endometrium using spatial transcriptomics, further suggesting the identity of stem-like *ALDH1A1*^+^ cells (Mareckova et al., 2024). Our analyses of ALDH^HI^ vs ALDH^LO^ human eutopic endometrial cells are in line with these previous findings, given that ALDH^LO^ cells displayed more luminal-like gene expression patterns, with prominent expression of cilia-related genes. ALDH^HI^ cells, on the other hand, displayed greater organoid formation capacity than ALDH^LO^ cells.

Endometrial stem cells are implicated in endometriosis pathogenesis due to previous studies that have identified abnormalities in the menstrual effluent of women with endometriosis. These include the presence of more basalis-like tissues in the effluent of women with endometriosis, containing peristromal muscular tissue markers (Leyendecker et al., 2002). More recent studies show that menstrual cells from patients with endometriosis express higher *SSEA*^+^*/SOX9*^+^ stemness markers (Hapangama et al., 2019). Furthermore, exome sequencing studies have identified matching DNA mutations to be present in the eutopic and ectopic endometrium, suggesting that endometrial tissue from women with endometriosis inherently has a selective growth advantage (Suda et al., 2018). Our studies show that eutopic ALDH^HI^ epithelial organoids have a higher organoid formation capacity and organoid formation rate than epithelial organoids established from ALDH^LO^ cells, supporting the hypothesis that endometrial stem cells within menstrual effluent are implicated in endometriosis. While further investigations are warranted to specify the role of ALDH^HI^ cell populations in endometriosis, our studies suggest that the presence of ALDH1A1+ cells in endometrial cells or menstrual effluent could be a biomarker for predicting a propensity for endometriosis.

Few mouse models have been used to further define and characterize stem and progenitor cells in the adult endometrium. Luminal epithelium, glandular structures, and stromal cells are induced to proliferate by the mitogenic potential of E2, while P4 induces differentiation in preparation for a pregnancy. At the end of the 4–5 day estrous cycle, the glandular folds are resorbed through processes involving apoptosis or autophagy (Dharma et al., 2001; Popli et al., 2022; Wood et al., 2007) (Popli et al., 2023). Single cell analyses of the postnatal uterus have identified key putative stemness genes that are critical for patterning of the endometrium (Fu et al., 2020; Spencer et al., 2023; Wu et al., 2017). Additionally, lineage tracing studies have identified that *Lgr5*^+^ *and Axin2*^+^ are long lived progenitors in the endometrium that are enriched in the crypts of endometrial glands and display stemness characteristics (Seishima et al., 2019; Syed et al., 2020). More recently, *Nestin*^+^ perivascular cells were shown to contribute to endometrial re-epithelialization in the adult mouse uterus (Li et al., 2025).

Previously, there was a lack of functional assays and lineage tracing studies to further characterize and confirm ALDH1A1^+^ endometrial stem cell populations. Using an inducible fluorescent ALDH1A1 reporter mouse, we characterized the presence and contribution of ALDH1A1^+^ cells throughout endometrial glandular development, the murine hormonal cycle, and in postpartum regeneration. By lineage tracing ALDH1A1^+^ cells at different developmental and adult timepoints, our results show that they give rise to cells that repopulate and persist in the glandular and luminal epithelium in the long-term. We hypothesized that the singly labeled cells observed from one day tracing experiments expanded in a clonal fashion during the various timepoints we measured. We note that the translation kinetics of the labeled cells following cre-mediated recombination may contribute to the limited labeling observed at PND8/PND15 and there is a potential for delayed labeling of cells between 24 and 72 hours of tamoxifen administration. However, the continuous increase in labeled cells at the subsequent timepoints favors our interpretation of clonal expansion as the primary explanation.

Endometrial glandular development in the mouse is a postnatal process that begins at approximately postnatal day 5 (PND5) and is completed by PND21 (Hayashi et al., 2011) (Vue et al., 2018). Glandular patterning in the postnatal period is attributed to signaling pathways that involve WNT/β-catenin, estrogen receptor (ESR1) signaling, BMPs, and other complex networks (Rizo et al., 2023) (Mericskay et al., 2004; Miller and Sassoon, 1998; Nanjappa et al., 2015). Transcriptomic analyses of the developing uterus have shown that genes involved in retinoic acid (RA) metabolism peak from PND0 to PND14 and then begin to gradually decline at PND28, suggesting that this process is critical in glandular patterning in the postnatal period (Wu et al., 2017). In line with these findings, our study showed that ALDH1A1 is highly expressed throughout the luminal uterine epithelium at PND7, with a gradual shift to the glandular crypts as glandular development progressed at PND14. Given the well-documented impacts of tamoxifen for lineage tracing studies, it is imperative to use doses of tamoxifen that will minimize estrogenic impacts and result in off-target effects (Rios et al., 2016). This often requires administration at doses that will achieve maximal recombination of the desired gene, while ensuring that the potential deleterious impacts of tamoxifen are minimized (Chen et al., 2023; Pimeisl et al., 2013). The cre/ERT2 tamoxifen inducible model is widely used to study uterine biology where it serves as a useful tool to interrogate the spatiotemporal impact of key genes, either through inactivation or for lineage tracing. Despite its widely documented utility across many tissue types and developmental timepoints, the use of tamoxifen and its impacts on the endometrium remain a limitation of our study, which we tried to address by implementing multiple timepoints, doses, and orthogonal assays in our experimental design.

To further address the impacts of ALDH1A1^+^ cells on glandular development, we used an inducible DT ablation model where DTR was conditionally expressed in the ALDH1A1-expressing cells. DT-mediated ablation of ALDH1A1^+^ cells was performed at PND7, led by the hypothesis that ablation of ALDH1A1^+^ cells would impair glandular development in adult mice. We analyzed uterine tissues of mice 56 days after administration of DT, where we indeed found fewer glands and reduced FOXA2 intensity in the mice with DT-mediated ablation of ALDH1A1^+^ cells. Similar results were obtained *in vitro*, where DT-mediated ablation of ALDH1A1^+^ cells caused organoid death. The observed partial ablation of glands that we found *in vivo* is consistent with the phenotype of ALDH1A1 KO mice, which are viable and fertile, suggesting that compensation by additional ALDH isozymes can rescue glandular development and function (Matt et al., 2005).

Interestingly, we also found the pattern of ALDH1A1 expression to be dynamic in the adult murine uterus, with restricted expression in the crypts during estrus and more diffuse staining throughout the luminal and glandular compartments in the diestrus phases. We confirmed that this dynamic ALDH1A1 expression was hormone driven by using an ovariectomized model treated with exogenous hormones. In this model, ovariectomy without hormones led to ALDH1A1 expression throughout the luminal uterine epithelium, while E2 and P4 treatment caused dynamic shifting of expression between the glandular crypts and luminal epithelium. Ovariectomized mice treated with 90-day E2 pellets, on the other hand, showed enriched ALDH1A1 in the glandular crypts with weak luminal epithelial staining, while the ovariectomized controls had strong ALDH1A1 expression throughout the luminal and glandular epithelium. Thus, it is possible that in the absence of hormones, the uterine epithelium takes on a more plastic state with both glandular and luminal cells displaying stemness qualities. Conversely, under the mitogenic actions of E2, when epithelial cell turnover is higher, ALDH1A1^+^ cells are restricted to the glandular crypts to remain as a reservoir for subsequent proliferative cycles.

The conversion of retinaldehyde to retinoic acid is driven by the ALDH1A enzymes, which are expressed in a spatiotemporally restricted pattern within the developing and adult endometrium (Vermot et al., 2000; Wu et al., 2017). In the adult, we observed that ALDH1A1 localized to the crypts of the endometrial glands of mice during the estrus phase and in ovariectomized mice supplemented with estradiol pellets (Supplemental Figure S1). The local synthesis and activity of retinoic acid via the retinoic acid receptor (RAR) may be critical for maintaining the stemness of adult endometrial epithelial cells, allowing for cellular proliferation and differentiation when exposed to estradiol at key phases of the cycle. This idea is supported by studies showing estrogen induces epithelial cell stratification in the vagina and cervix and increases expression of RAR (and its heterodimeric partner, RXR) in basal epithelial cells, suggesting that the two pathways of estrogen and retinoic acid signaling converge (Celli et al., 1996; Tannous-Khuri and Talmage, 1997). Further, recent studies using uterine conditional ablation of RARA/RARB/RARG with the progesterone receptor cre show that these mice develop excessive stratification of the uterine luminal epithelium upon RAR ablation (Yin et al., 2025). Because improper epithelial cell stratification is counteracted by administration of Fulvestrant, a potent estrogen receptor antagonist, the authors conclude that RA/RAR signaling antagonizes E2/ER action and is required for epithelial cell fate maintenance in the adult. Thus, it is plausible that ALDH1A1 activity, through its impact on RA/RAR activity and E2/ER signaling, is driving endometrial cell differentiation and maintaining a reservoir of quiescent stem-like cells in the endometrial epithelium.

Additionally, our previous studies showed that conditional ablation of the downstream effectors of the transforming growth factor β (TGFβ) signaling pathway in the uterus, SMAD2 and SMAD3, disrupted epithelial cell homeostasis, leading to excessive estrogen-dependent cell proliferation, endometrial tumors, and disrupted retinoic acid metabolism (Kriseman et al., 2019; Kriseman et al., 2023; Monsivais et al., 2019). Hence, it is also plausible that TGFβ/SMAD2/3 are critical for integrating the RA/RAR-dependent antagonism of E2 action into the epithelium, thereby directing proliferation and differentiation programs in the adult endometrium. The exact mechanisms controlling this antagonism, however, are not yet known, and likely involve paracrine signaling networks between the endometrial stroma and epithelium. Whether ALDH1A1 and other ALDH isozymes contribute to similar proliferative and stemness programs in the stroma remains to be evaluated.

We also found ALDH1A1^+^ stromal cells were more prevalent when tracing began in adult mice. Other studies have shown that mesenchymal cells contribute to endometrial regeneration in the postpartum phase or after induced menses through a process of MET (Cousins et al., 2014; Kirkwood et al., 2022; Li et al., 2025). Similarly, lineage tracing studies have shown that MET is an active process and contributes to epithelial cell regeneration in the post-partum phase (Huang et al., 2012; Patterson et al., 2013). Although this is an area of active investigation in the field, with some contradicting reports, it is plausible to hypothesize that endometrial tissue has the capacity to undergo wound-healing and regeneration via several mechanisms (Ang et al., 2023; Ghosh et al., 2020). The process of MET in wound healing is widely documented in other organs, such as the kidney, liver and lung, where MET is associated with depletion of the resident epithelial cell pool (Bi et al., 2012; Niayesh-Mehr et al., 2024; Zeisberg et al., 2005). This prompted us to determine whether the stromal ALDH1A1^+^ cells were contributing to epithelial regeneration in the postpartum phase. Analyses of PPD3 regenerating endometrium showed a greater number of cells expressing VIM/CK8/RFP compared to the PPD5 endometrium, suggesting that the presence of these transitional cells was more frequent at PPD3. We noted that not all the Vim^+^/CK8^+^ cells expressed ALDH1A1^+^, suggesting that the cells marked by all three represent a subpopulation of cells that contribute to MET in the postpartum phase. However, because at the time of labeling both epithelial and stromal cells express ALDH1A1, this does not exclude the possibility that the transitional VIM/CK8/RFP cells we observed were undergoing EMT and not MET. We also noted that a majority of ALDH1A1^+^ cells were localized to the active areas of endometrial regeneration near the placental detachment sites at PPD1 with a pronounced expression in the sub-epithelial stromal cells. As regeneration progressed, we continued to observe ALDH1A1^+^ cells in the stromal compartment within the placental detachment sites at PPD3 and PPD5, with a progressive, but not statistically significant, increase in ALDH1A1^+^ epithelial cells. Collectively, our data demonstrate that ALDH1A1^+^ lineage cells participate in the restoration of endometrial architecture and functional compartments in the postpartum phase, even if their direct contribution is transient. Future detailed and mechanistic studies will be necessary to fully characterize their role in this process and their long-term consequence in postpartum regeneration.

Overall, our lineage tracing, ablation, and regeneration models show that ALDH1A1^+^ endometrial cells display characteristics of an adult stem cell. Organoid formation assays in ALDH^HI^ vs. ALDH^LO^ endometrial cells from both human and mice support these findings and place the activity of ALDH1 enzymes as central regulators of regenerative potential in the endometrium. This is also observed in the scRNAseq analyses of the adult cycling mouse uterus, where ALDH1A1^+^ cells that lacked proliferative markers clustered with *Lgr5* and *Axin2*-expressing cells and displayed a trajectory of EpSCs giving rise to both glandular and luminal cells. Clustering with this population of EpSCs were *Susd2*^+^ cells, which are well-characterized mesenchymal progenitors that are enriched in the perivascular regions of the human endometrium (Darzi et al., 2016; Khanmohammadi et al., 2021). The presence of *Susd2*^+^ cells, while unexpected in an epithelial stem cell niche, could indicate the presence of a transitional mesenchymal or perivascular cell that is differentiating into epithelium. Evidence for both mesenchymal and *Nestin2*^+^ pericytes have been recently described in the mouse endometrial epithelium (Kirkwood et al., 2022; Li et al., 2025). By integrating with scRNA profiles of EpSCs characterized in previous studies (Padilla-Banks et al., 2023; Winkler et al., 2024), this population of cells and datasets can be used to identify and characterize additional stem cells in the adult endometrial epithelium.

Additionally, our studies in human endometrium extend our characterization of ALDH1A1 as an adult endometrial stem cell marker and emphasize the importance of ALDH1A1^+^ in the regenerative potential of the endometrium. The conserved hormonal responses between human and mouse endometrium support the hypothesis that cycles of proliferation, differentiation, and regression, whether through resorption/autophagy in mice or menstrual breakdown in humans, are governed by concerted growth factor signaling and dedicated stem cell populations with the capacity to expand and differentiate across repeated cycles of repair. Our detailed studies in both mouse and human models indicate that ALDH1A1^+^ cells represent a dedicated cell type within the endometrium with the potential to drive repair during adulthood. Collectively, these findings advance our understanding of the mechanisms that contribute to endometrial cycling and regeneration throughout the reproductive lifespan.

## Methods

### Human sample collection

Tissues were collected from patients after obtaining informed written consent and following the guidelines as approved by the Baylor College of Medicine IRB protocol (H-21138). Tissues and any cells derived from them were de-identified prior to use to ensure patient confidentiality guidelines. Donor age and clinical information is reported in Supplemental Table S5.

### Animal ethics statement

All animal procedures were approved by the Institutional Animal Care and Use Committee (IACUC) of Baylor College of Medicine (BCM) and guidelines established by the National Institutes of Health Guide for the Care and Use of Laboratory Animals. All the mice were housed under standard conditions of a 12 h light/dark cycle in a vivarium that maintained a controlled ambient temperature of 70 °C ± 2 °C and a relative humidity of 20–70%.

### Mouse models and genotyping

The *Aldh1a1*^cre/ERT2^ mice were obtained from Dr. Raj Awatramani, Department of Neurology, Northwestern University Feinberg Medical School (Azcorra et al., 2023), while the Ai9 tdTomato mice (B6.Cg-Gt(ROSA)26Sor^tm9(CAG-tdTomato)Hze^/J) were provided by Dr. Stephanie Pangas, Department of Pathology and Immunology, Baylor College of Medicine. The ROSA26iDTR mice (C57BL/6-*Gt(ROSA)26Sor*^*tm1(HBEGF)Awai*^/J, JAX strain # 007900) were purchased from the Jackson Laboratory. All experimental mice were either homozygous for Ai9 tdTomato or iDTR. Genotyping was performed using DNA extracted from 2–3 mm tail snips. Tail samples were digested in 200 μL of 50 mM NaOH at 95°C for 30 minutes. Following digestion, 100 μL of 1 M Tris-HCl (pH 8.0) was added to neutralize the solution, and the mixture was centrifuged at maximum speed for 5 minutes to pellet any debris. The supernatant containing the isolated DNA (1–2 μL) was used as a template for PCR amplification. Amplification was performed using amfiSure PCR Master Mix (GenDepot) with the primer sequences provided in Supplementary Table S4 and following the cycling conditions detailed in Supplementary Figure S7.

### Aldh1a1 lineage tracing mouse experiments

4OH-Tamoxifen (Sigma, H7904) and tamoxifen (Sigma, T5648) powders were initially dissolved in 100% ethanol and then diluted in corn oil at a concentration of 10 mg/mL. For *Aldh1a1* lineage tracing at early stage, *Ai9/Ai9* and *Ai9/Ai9*; *Aldh1a1*^*cre/ERT2*^ female mice received a single intraperitoneal (IP) injection of either 4OH-Tamoxifen at 0.5 μg/g body weight on postnatal day 7, 8, or tamoxifen at 0.15mg/g body weight on postnatal day 14 or 21. Mouse uteri were then harvested on postnatal days 8,14, 15, 21, 28, and 56 respectively. For *Aldh1a1* lineage tracing across estrous cycles, *Ai9/Ai9*; *Aldh1a1*^*cre/ERT2*^ female mice at the age of 6 weeks in estrus were injected with a single dose of tamoxifen at 0.15mg/g body weight. Their uteri were then collected either in the next estrus or estrus one month later. To trace ALDH1a1+ cells in endometrial remodeling and regeneration during pregnancy, *Ai9/Ai9*; *Aldh1a1*^*cre/ERT2*^ female mice were given a single dose of tamoxifen at 0.15mg/g body weight at the age of two months and then mated with WT male adult mice one week later. Mouse uteri were collected on postpartum days (PPD) 1, 3 and 5.

### Surgeries and hormone treatments

Six-week-old female mice were ovariectomized and given a two-week period to ensure the complete clearance of residual ovarian hormones. The ovariectomized mice received two doses of estradiol-17β (E2, 100ng/mouse, Sigma, E8875) through subcutaneous injection. After two days’ rest, the mice were randomly divided into four groups: Vehicle group received 4 doses of sesame oil, P4 group received 4 doses of progesterone (P4, 1mg/mouse, Sigma, P0130), E2 group received 3 doses of sesame oil followed by one dose of E2 (100ng/mouse), P4+E2 group received 3 doses of P4 (1 mg/mouse) followed by one combined dose of 100ng E2 and 1mg P4. The uterine horns from these four groups were collected 15 hours after receiving the last dose. Six-week-old female mice were ovariectomized and implanted with a placebo or estradiol pellet (17β-ESTRADIOL, 0.025 mg, 90 days, Innovative Research of America, NE-121) and their uteri were collected after 90 days.

### RNA extraction and quantitative real-time PCR

Organoids or tissue samples were homogenized in Trizol reagent (Life Technology, 15596018) and total RNA was extracted using the Direct-zol RNA MiniPrep kit (Zymo Research, R2052) according to the manufacturer’s protocol. Reverse transcription was performed using the qScript cDNA SuperMix (Quantabio, 95048–100) with 200ng of total RNA as template, following the manufacturer’s instructions. cDNA was diluted three times in water. Quantitative real-time PCR (qPCR) was carried out on a BioRad CFX Real-Time PCR System using SYBR Green Master Mix (Life Technology, 4364346). Each 10 μL reaction contained 10 μL of cDNA, 0.5 μM of each gene-specific primer, and 1X SYBR Green mix. The PCR primers are listed in Supplementary Table S4. All reactions were performed in duplicate with three biological replicates. Gene expression levels were normalized to the housekeeping gene *Rpl17 or Hprt* and relative quantification was calculated using the 2^(−ΔΔCt) method (Schmittgen and Livak, 2008). Data were presented as the mean fold change ± SEM and analyzed using a two-tailed *t*-test in GraphPad Prism.

### Mouse endometrial epithelium dissociation for organoids and single-cell RNA sequencing (scRNAseq)

Epithelial cells were isolated from mouse endometrial tissue using a combination of mechanical and enzymatic dissociation as previously described (Tang et al., 2023b). Briefly, uterine horns were dissected from six-week-old female wild-type (WT) mice at the estrus stage, as confirmed by vaginal cytology, and were then cut into small fragments (4–5 mm) using sterile scissors. Tissue fragments were moved to a digestion solution containing 1% Trypsin (Sigma, T1426) in HBSS (ThermoFisher, 14170112) and incubated at 37°C for 45 minutes. Following incubation, the uterine fragments were moved to a 35 mm tissue culture plate containing 1 mL of Dulbecco’s Phosphate-Buffered Solution (DPBS), where epithelial sheets were separated mechanically from the uterine tubes using a 1 mL pipette. The epithelial sheets were then collected and pelleted by centrifugation at 2000 rpm for 5 minutes at 4 °C. The pellets were resuspended in HBSS containing collagenase I (5mg/mL, Sigma, C0130) and DNase I (0.2mg/mL, Sigma, DN25) and subsequently filtered through 100 μm cell strainers. The red blood cells were lysed in 0.2% NaCl for 20 seconds followed by 1.6% NaCl. The resulting single cells were then used for organoid culture or live cell sorting using DAPI.

### Human endometrial epithelium dissociation

Endometrial organoids were established from human fresh endometrial tissue samples obtained with informed consent following ethical approval. Tissue samples were minced into small fragments and enzymatically digested using a combination of 5mg/mL collagenase I (Sigma, C0130) and 0.2mg/mL DNase I (Sigma, DN25) in HBSS (ThermoFisher, 14170112) for 1 hour at 37°C with gentle agitation. The resulting cell suspension was filtered through a 100 μm cell strainer to remove undigested tissue, and the filtrate was centrifuged at 2000 rpm for 5 minutes to pellet the cells. The red blood cells were lysed in 0.2% NaCl for 20 seconds followed by 1.6% NaCl. The resulting single cells were then used for organoid culture.

### Mouse and human endometrial organoid culture

The isolated mouse epithelial cells were resuspended in ice-cold Matrigel (Corning, 354230) and seeded as 30 μL droplets onto 12-well culture plates. After allowing the Matrigel to solidify at 37°C for 15 minutes, the mouse organoid culture medium, composed of Advanced DMEM/F12 (Life Technologies, 12634010) supplemented with 1× B27 (Life Technologies, 12587010), 1× N2 (Life Technologies, 17502048), 100 μg/mL primocin (Invivogen, ant-pm-1), 1.25mM N-Acetyl-L-cysteine (Sigma, A9165), 2mM L-glutamine (Life Technologies, 25030024), 50 ng/mL EGF (PeproTech, AF-100–15), 100ng/mL FGF-10 (PeproTech, 100–26), 50ng/mL HGF (PeproTech, 100–39), 10% Noggin (BCM Digestive Diseases Center), 10% R-spondin (BCM Digestive Diseases Center), 10% WNT3a (BCM Digestive Diseases Center), 10nM Nicotinamide (Sigma, N0636), and 10 μM Y-27632 (ROCK inhibitor, Sigma, Y0503), was gently added to each well. Human isolated epithelial cells were also resuspended in ice-cold Matrigel (Corning, 354230), seeded as 30 μL droplets onto 12-well culture plates, and fed with human organoid culture medium containing complete mouse organoid medium supplemented with 1 μM A83–01 (Tocris, 2939). Organoids were maintained at 37°C in a humidified incubator with 5% CO_2_, and the medium was refreshed every 2–3 days. Organoid growth and morphology were monitored under an inverted microscope, and passaging was performed every 7–10 days by mechanically disrupting the matrigel and reseeding them in fresh matrigel. For experimental assays, organoids were dissociated into single cells using Accutase cell dissociation reagent (Life Technologies, A1110501), followed by further processing or analysis as required.

### ALDEFLUOR assay and fluorescence-activated cell sorting

Cell sorting based on aldehyde dehydrogenase (ALDH) activity was performed using the ALDEFLUOR kit (StemCell Technologies, 01700) according to the manufacturer’s instructions. Briefly, single-cell suspensions were prepared from cultured organoids or dissociated tissues and resuspended in ALDEFLUOR assay buffer at a concentration of 1 × 10^6 cells/mL. The cell suspension was divided into two aliquots: one for the experimental sample and the other for the negative control containing the ALDH inhibitor diethylaminobenzaldehyde (DEAB). To each tube, activated ALDEFLUOR reagent was added, and the samples were incubated at 37°C for 30–45 minutes in a CO2 incubator. During this incubation, cells with high ALDH activity converted the ALDEFLUOR substrate into a fluorescent product that accumulates intracellularly. Following incubation, cells were centrifuged at 2000 rpm for 5 minutes, resuspended in fresh ALDEFLUOR assay buffer, and kept on ice until sorting. Flow cytometry was performed using a BD FACSAria sorter equipped with appropriate filters to detect ALDH-dependent fluorescence (FITC channel) by the Cytometry and Cell Sorting Core at Baylor College of Medicine. Cells treated with DEAB served as a baseline to define the ALDH-negative population (ALDH^LO^), ensuring accurate gating of ALDH-positive cells (ALDH^HI^). ALDH^LO^ and ALDH^HI^ cell populations were collected in culture medium supplemented with 10% fetal bovine serum (FBS) to preserve viability and immediately processed for downstream applications. All flow cytometry data were analyzed using FlowJo software to confirm sorting accuracy and purity.

### Organoid Formation Assay

Sorted ALDH^LO^ and ALDH^HI^ cells from human organoids or pools of mouse endometrial epithelial cells at the estrus stage were directly seeded in equal numbers onto 40μL Matrigel domes in a 24 well plate and cultured in organoid medium for ~2 weeks. To perform organoid formation assays with both the mouse and human organoids, organoids were first resuspended in ice-cold Advanced DMEM/F12 to break up organoids from the Matrigel. The organoids were spun down at 600 ×g for 5 minutes, the supernatant was removed, and the Matrigel and organoid layer remaining were again resuspended 2–3 more times until the organoids were separated from the Matrigel layer. Next, the Matrigel layer was removed, and the organoid pellet was resuspended in 5 mL of StemPro Accutase Cell Dissociation Reagent (ThermoFisher Scientific, A1110501). The Accutase-organoid mixture was incubated on a shaker in the 37 °C tissue culture incubator for 30–40 minutes until a single-cell suspension was achieved. The suspension was then spun down at 600 ×g for 5 minutes and filtered through a sterile 40 *μ*m filter (Corning, 352340) followed by a 20 *μ*m sterile filter (PluriSelect, 43–50020-03) to ensure only single cells remain. The flow through was spun-down as previously mentioned and resuspended in a cell-counting volume. A small volume of the suspension was then mixed 1:1 with Trypan-Blue (Gibco, 15250061) to count alive cells with a manual hemocytometer. Cells were resuspended in a ratio of 100 cells/1 *μ*L of Matrigel and plated in triplicate with one 40 *μ*L Matrigel dome plated in the center of the well in a 24-well plate. The Matrigel domes were then allowed to solidify in the 37 °C tissue culture incubator for 10 minutes before 750 *μ*L of complete organoid media (+A83–01 for human, −A83–01 for mouse) with 10 *μ*M of Y-27632 dihydrochloride (Sigma, Y0503) was added. Media was changed every 2 days, and only the first two days of media had the addition of Y-27632 to help the organoids reconstitute. Wells were imaged on Day 7 with the Yokogawa CV8000 or the BioTek Cytation 5. Images were then tiled and stacked to create z-projections of the full Matrigel dome plated. For the human organoids, organoid quantification and analysis were completed using a trained-AI model on the final z-projection images through BioDock (Biodock, AI Software Platform. Biodock 2024. Available from www.biodock.ai.) For mouse organoids, organoid quantification and analysis were completed manually using ImageJ.

### *In vitro* and *in vivo* Aldh1a1 ablation

Diphtheria toxin (DT, Sigma, D0564) was resuspended in sterile water at a concentration of 1mg/mL. Endometrial epithelial cells were isolated from the uteri of six-week-old female ROSA26^DTR/DTR^ and ROSA26^DTR/DT*R*^; *Aldh1a1*^*cre/ERT2*^ mice at the estrus stage and cultured in organoid medium for one week to establish organoids. After the initial culture, the organoids were passaged with an equal number of cells seeded into 40μL Matrigel domes in a 24 well plate and further cultured for an additional week. On day 7, the organoids were treated with 0.01mg/mL 4OH-T followed by exposure to 0.25 μg/mL DT on day 9. The organoids were harvested on day 10, fixed in 4% paraformaldehyde, and subsequently analyzed for apoptotic marker, cleaved caspase-3. To ablate ALDH1A1+ cells in the mouse endometrium, ROSA26^DTR/DTR^ and ROSA26^DTR/DT*R*^; *Aldh1a1*^*cre/ERT2*^ mice were administered a single dose of 4OH-Tamoxifen at 0.5 μg/g body weight on postnatal day 7 (PND7), following by a single dose of DT at 16.6 μg/g body weight on PND10. Mouse uteri were collected on PND 56 and analyzed to evaluate glandular development.

### Immunohistochemistry and immunofluorescent staining

Mouse uteri were fixed in 10% neutral-buffered formalin for 24 hours and then stored in 70% ethanol. Human or mouse organoids were fixed in 4% paraformaldehyde for 24 hours, mounted in histogel processing gel (Thermo Scientific, HG-4000–012), and stored in 70% ethanol. Tissue samples were processed and embedded in paraffin using standard histological procedures. Sections of 5 μm thickness were mounted onto adhesive microscope slides. Prior to staining, the slides were deparaffinized in xylene, rehydrated through a series of decreasing concentrations of ethanol, and rinsed in distilled water. Antigen retrieval was performed by heating the slides in a citrate buffer (pH 6.0) using a microwave. For IHC staining, sections were incubated in 3% hydrogen peroxide for 10 minutes to quench endogenous peroxidase activity. Non-specific binding was blocked with 3% bovine serum albumin (BSA) in Tris-buffered saline with Tween 20 (TBST) for 60 minutes at room temperature. The sections were then incubated overnight at 4°C with primary antibodies (Supplementary Table S4) diluted in blocking buffer according to the manufacturer’s recommendations. Following this, the slides were washed in TBST and incubated with a biotinylated secondary antibody (Supplementary Table S4) for 60 minutes, followed by the incubation with the horseradish peroxidase (HRP)-conjugated streptavidin complex (Vector Laboratories, PK-6100). Visualization was achieved using 3,3'-diaminobenzidine (DAB, Sigma, D5637) as the chromogen, which produced a brown precipitate at the site of antibody binding. The sections were counterstained with Harris hematoxylin (Sigma# HHS32) to visualize nuclei, dehydrated, and mounted with Permount mounting medium (Fisher Scientific, SP15). Negative controls were prepared by omitting the primary antibody. All stained slides were scanned at 40× by the Digital Pathology Service from the BCM Department of Pathology & Immunology. For IF staining, after antigen retrieval sections were permeabilized with 0.1% Triton X-100 in TBST for 10 minutes to allow intracellular antibody access. Non-specific binding was blocked with 3% bovine serum albumin (BSA) in Tris-buffered saline with Tween 20 (TBST) for 60 minutes at room temperature. The sections were then incubated overnight at 4°C in a humidified chamber with primary antibodies (Supplementary Table S4) diluted in blocking buffer according to the manufacturer’s recommendations. The next day, the sections were washed and incubated with secondary antibodies conjugated to fluorophores (Supplementary Table S4) for 1 hour at room temperature, protected from light. Nuclei were counterstained with 4',6-diamidino-2-phenylindole (DAPI) for 5 minutes, followed by thorough washing with TBST. The coverslips were mounted onto glass slides using Vectasheild anti-fade mounting medium (Vector Laboratories, H-1000) to preserve fluorescence. Slides were stored in the dark at 4°C until imaging. Negative controls were prepared by omitting the primary antibody. All fluorescently labeled slides were evaluated and imaged at the Optical Imaging and Vital Microscopy Core Facility Laboratory at Baylor College of Medicine using an LSM880 confocal microscope.

### Transcriptomic Profiling of Human Eutopic Organoids by RNA Sequencing

Total RNA was extracted from cultured organoids or tissue samples using the DirectZol kit (Zymo Research, R2052) following the manufacturer’s protocol, with an additional on-column DNase I digestion step to remove genomic DNA contamination. Sorted ALDH^Hi^ and ALDH^LO^ organoids from eutopic endometrium samples of three patients with endometriosis were analyzed. Three wells of a 12-well plate of ALDH sorted organoids containing two 30μl domes of Matrigel from each patient were pooled (n=3 patients) and total mRNA was extracted using DirectZol Kit from Zymo. RNA with a high integrity (RIN >8) was used for library preparation using the Illumina TruSeq RNA Library Prep Kit and sequenced with Illumina Novaseq (Novogene, Inc., Sacramento, CA). Differentially expressed genes (DEGs) between the ALDH^HI^ and ALDH^LO^ groups were calculated using DEseq2 (version 1.42.1) with a log2FC > 1 and < −1 and an adjusted *p* value < 0.05. The DEGs were plotted and visualized using SRplot (Tang et al., 2023a). Biological process gene ontologies of the separated up-regulated or down-regulated DEGs were identified using enrichGO in clusterProfiler (Yu et al., 2012) (version 4.10.1) and then replotted using SRplot. Sequencing data of all human organoid samples are available at GSE294342.

### Transcriptomic Profiling and Comparison of Mouse Endometrial Organoids by RNA Sequencing

Total RNA was extracted from cultured mouse endometrial organoids at passage 3–5 using the DirectZol kit (Zymo Research, R2052) following the manufacturer’s protocol, with an additional on-column DNase I digestion step to remove genomic DNA contamination. Endometrial epithelial cells from ~13 WT (randomly cycling) were subjected to the ALDEFLUOR assay and sorted into ALDH^LO^ and ALDH^HI^ populations. After sorting, the cells were plated and expanded for 3–5 passages. The experiment was repeated three different times. The ALDH^LO^ and ALDH^HI^ organoids were collected in Trizol, and profiled using RNAseq. RNA with a high integrity (RIN >8) was used for library preparation using the Illumina TruSeq RNA Library Prep Kit and sequenced with Illumina Novaseq (Novogene, Inc., Sacramento, CA). DEGs between the mouse ALDH^HI^ and ALDH^LO^ groups were calculated using DEseq2 (version 1.42.1) with a log2FC > 1 and < −1 and an adjusted *p* value < 0.005. The DEGs were plotted and visualized using SRplot (Tang et al., 2023a). Syed et al.’s AXIN2^HI^ RNA sequencing data was acquired from Series GSE140222 where the gene list was filtered according to log2FC > 1 and < −1 and an adjusted *p* value < 0.005 to identify significantly up and down genes to compare to the ALDH^HI^ gene lists. Overlap of the up and down genes between the ALDH and AXIN2 lists was done with Gene List Venn Diagram available at https://www.bioinformatics.org/gvenn/. Sequencing data of the ALDH^HI^ and ALDH^LO^ mouse endometrial organoids are available at GSE294342.

### Single-cell RNA sequencing (scRNAseq)

Endometrial epithelial cells were isolated from ~6 adult WT mice uteri at the stages of estrus and diestrus as described above (“Mouse endometrial epithelium dissociation for organoids and single-cell RNA sequencing.”) Epithelial cells were viably frozen in 90% FBS and 10% DMSO until the day of analysis. Single viable cells from the 6 adult WT mice were obtained by sorting on a BD FACS DIVA using a 100μm nozzle, low pressure, and using DAPI and capturing in 100% FBS to increase viability. After the sorting, live cells we centrifuged and resuspended in PBS to adjust the concentration to ~ 1600 cells/μL and a targeted cell recovery of 20000 total cells. Single-cell RNA sequencing libraries were prepared using the Chromium Single Cell 3' Reagent Kits v4 (10x Genomics) according to the manufacturer’s instructions. In brief, single-cell suspensions were loaded onto the Chromium Controller to generate Gel Bead-In-Emulsions (GEMs), followed by reverse transcription and barcoded cDNA-library construction via T100 Thermal Cycler (Bio-Rad Laboratories). The resulting libraries were assessed and confirmed to pass quality controls using the Agilent TapeStation system with High Sensitivity D1000 ScreenTape assays (Agilent Technologies). The sequencing was performed on the Illumina NovaSeq X platform (Novogene, Inc) using paired-end 150 bp reads, targeting approximately 24,000 reads per cell to ensure sufficient coverage for robust downstream analysis. Raw sequencing data were processed using Cell Ranger 9.0.1. The raw reads were aligned to the GRCh38 reference genome, and feature-barcode matrices were subsequently generated. Raw count matrices were imported into Seurat 5.2.0 for filtering, scaling, normalization, dimensional reduction, and clustering. The analysis of trajectory inference was performed using Slingshot 2.16.0 for constructing developmental lineages and identifying dynamic marker genes. The single cell RNA sequencing data for the enriched epithelial and stromal populations from mouse uteri in both estrus and diestrus phases are available at GSE294342.

### Statistics and analysis

Statistical analyses were performed using GraphPad Prism. Data are presented as mean ± standard error of the mean (SEM) or standard deviation (SD), as indicated in the figure legends. For comparisons between two groups, unpaired or paired Student’s *t*-tests were used for normally distributed data, while Mann-Whitney U or Wilcoxon signed-rank tests were applied for non-parametric data. For multiple group comparisons, one-way or two-way ANOVA was performed, followed by appropriate post hoc tests to adjust for multiple comparisons. A p-value < 0.05 was considered statistically significant, and all statistical tests were two-tailed unless otherwise specified. Outliers were identified and excluded only if justified by experimental or technical reasons. All graphs were generated using GraphPad Prism and raw data were maintained for reproducibility and transparency.

## Supplementary Material

This is a list of supplementary files associated with this preprint. Click to download.

• ALDH1A1SupplementalData5.6.26revisedfinal.pdf

• SupplementalTableS1MouseOrganoidHivsLov2.xlsx

• SupplementalTableS1MouseOrganoidHivsLo.xlsx

• SupplementalTableS5.xlsx

• SupplementalTableS5revised.xlsx

## Data Availability

Sequencing data are available in the Gene Expression Omnibus under the superseries accession number (GSE294342, secure reviewer token, sdmtuskkbxorlql).
